# Assessing the Correlation between Malaria Case Mortality Rates and Access to Health Facilities in the Malaria Region of Vhembe District, South Africa

**DOI:** 10.1155/2020/8973739

**Published:** 2020-12-02

**Authors:** Ryno Harm Coetzer, Abiodun Morakinyo Adeola

**Affiliations:** ^1^Department of Geography, Geoinformatics and Meteorology, Centre for Environmental Studies, University of Pretoria, Private Bag X20, Hatfield 0028, South Africa; ^2^South African Weather Service, Private Bag X097, Pretoria 0001, South Africa; ^3^UP Institute for Sustainable Malaria Control, School for Health Systems and Public Health, University of Pretoria, Pretoria 0002, South Africa

## Abstract

**Background:**

Local villages in the Vhembe district of South Africa have experienced high malaria infection rates and a high variability of malaria case mortality rates over the past 20 years. This research project sets out to determine if specific socioeconomic factors have influence on the varying malaria case mortality rates.

**Methods:**

The study used existing malaria records of all reported malaria cases in the Vhembe district between 1998 and 2017. The data set was sampled using maximum variation sampling combined with a stratified sampling approach to select the source locations with the highest reported variations in malaria case mortality. The number of medical facilities used, distances to the medical facilities, and proximity to significant water sources were subsequently spatially and statistically analysed for potential correlations between these factors and the malaria case fatality rates of the source locations.

**Results:**

Within the period of study, a total of 57,974 malaria infections were reported from 850 source locations across the villages and neighbourhoods. The result of the sampling methods gave 30 source locations with highest reported variations in malaria case mortality. The statistical analysis indicated a significant negative correlation between the case mortality rates and the number of medical facilities used, the number of infections reported, and the maximum and mean distances travelled to the medical facilities used. In addition, the analysis indicated a positive correlation between the minimum distances travelled to the medical facilities used and the case mortality rates. The spatial analysis supported the majority of the findings from the statistical analysis. Proximity to significant water bodies was not found to have any significant impact on case mortality rates.

**Conclusion:**

The results suggested that malaria patients from larger communities, those who had financial or other means to consult more advanced facilities, or those with a larger variety of services had a significantly lower risk of mortality. The findings of this study could assist societies and authorities in mitigating the negative effects of malaria infections on human life expectancies through improved socioeconomic development.

## 1. Introduction

Malaria is a significant vector-borne disease that influences the global population and places a detrimental health care burden on many communities globally [1]. The spatial distribution of malaria infections is significantly influenced by climate conditions and the ability of communities to prevent and treat the disease [2]. Previous studies have investigated the impact of climate change on vector-borne diseases with significantly conclusive results, indicating that climate change can have a major impact on the spreading of vector-borne diseases, particularly malaria [3]. This has been recorded in multiple areas, including the Northwest Frontier Province of Pakistan, Eastern Africa, and South Sudan, where a direct link was found between humidity increases due to climate change and the instances of malaria infections and death [4, 5]. Parham et al. [6] conducted intensive research to determine the link between malaria cases and climatic factors through a semi-parametric econometric model. The findings indicated that a marginal change in temperature and precipitation could result in a substantial change in the number of malaria cases, and further research was recommended, which indicates that spatial scales of available climate prediction need to be overlaid with socioeconomic data on a local scale to determine if infrastructure and other economic development aspects will affect the level of anticipated transmissions. Hence, the authors concluded that a panache concept needs to be explored and applied at an environmental and social level to further understand environmental and socioeconomical changes and their effects on mankind. In addition, Frank et al. [7] used a spatiotemporally validated model of *Plasmodiom falciparum* malaria transmission in Africa and analysed this against the Hadley Centre global climate change model to predict the potential effect of climate change on malaria transmission in Africa. The model suggested that malaria distribution could increase by 5%–7%, mainly owing to altitudinal spreading with limited change expected in the latitudinal spreading of the disease. According to Caminade et al. [8], studies attempting to forecast future malaria transmission according to climate change predictions could contribute significantly to understanding the future risks posed by malaria transmissions on a global scale. The study highlights the necessity to understand other socioeconomic factors and trends to integrate with climate change malaria transmission models, which will provide for an even more accurate risk assessment.

Several studies have investigated the relationship between socioeconomic factors and the rate of malaria infections. For instance, Christopher et al. [9] using a structured questionnaire investigated the link between socioeconomic variables such as demographic factors, including age; sex; complexion; body odor; clothing; marital status; family size; socioeconomic status, primarily focused on income groups and education levels; knowledge; awareness and education on malaria-related topics; and malaria infection rates. The study concluded that housing types, access to medical facilities, income levels, and malaria awareness could be associated with the occurrence of malaria. In addition, the study indicated that exposure to mosquito bites and the use of bed nets were positively associated with the occurrence of malaria. In another study, Kavita et al. [10] conducted research that links socioeconomic factors with malaria-related features in northern Tanzania using structured questionnaires administered to randomly selected households. The study focused on the effect of socioeconomic characteristics of an individual's response (treatment-seeking behaviour) to malaria infection as opposed to actual malaria infections, which Christopher et al. [9] researched. The questionnaire gathered information on the following socioeconomic aspects: knowledge of malaria vectors and larva control; ownership and use of malaria prevention tools; wealth of the household head; exposure to malaria prevention programmes, for example, the distribution of bed nets; formal education levels; and the distance from health care centres. The study found that the distance to health centres was the greatest factor influencing the willingness to treat infections, while the education levels of household heads were positively associated with efforts to prevent infections.

On the other hand, a snap literature review of the research that investigated potential relationships between socioeconomic factors and malaria case mortality rates revealed that limited knowledge of the topic is currently available; however, there are several studies that indirectly provide an insight on the topic. A study by Lowassa et al. [11] investigated the impact of access to primary health care on the severity of malaria morbidity, with a specific focus on children up to five years of age. The study aimed to understand the effectiveness of providing primary health care as a tool to minimize the effects of malaria on vulnerable communities. The study concentrated primarily on the relationship between travel times to the nearest primary health care facility and the incidence of hospitalization due to malaria infections. The result indicated that access to primary health care facilities could reduce the burden of malaria disease by up to 66%, and that an increase from ten minutes to two hours in travel time to health care facilities could double the likelihood of hospitalization from malaria infection. Similarly, O”Meara et al. [12] conducted research on the pattern of malaria-related deaths in patients between the ages of six and 31 years across 18 villages surrounding Centre de Recherche en Santé de Nouna in the Nouna Health District in northwestern Burkina Faso. The research was conducted primarily through field observations by village workers over a six month period. The analysis revealed that a lack of appropriate second-line treatment at formal health care facilities was one of the leading causes of malaria-related deaths in the area. In addition, the study revealed that ease of access to medical facilities had a significant impact on a patient's willingness to seek medical attention, which in turn could result in fatalities.

Muller et al. [13] found that the rollout of national programmes that enable community health workers to support vulnerable communities can reduce malaria mortality rates by between 36% and 63% in sub-Saharan Africa. This was indicated through a study that investigated and compared the changes in malaria case mortality rates before and after community health worker programmes were implemented. The data were obtained through researching existing databases of published and unpublished studies for community health worker programmes and their effectiveness in communities [13].

Based on the suggestion by Caminade et al. [8], this study aims to build on current malaria risk assessment models by investigating how specific socioeconomic factors are able to influence malaria case fatality rates. This could support decision-makers to not only forecast malaria transmission patterns but also understand and mitigate the malaria case fatalities, which can be considered to be the most significant impact arising from changing malaria transmission patterns. Therefore, the primary focus of this research is the relationship between specific socioeconomic factors and malaria case mortality rates. The rationale is that climate change could lead to a redistribution of malaria infections in South Africa, making it crucial to understand all the measures that could mitigate the effects of infections and potential fatalities in vulnerable societies.

## 2. Methodology

### 2.1. Study Area

**Figure 1:**
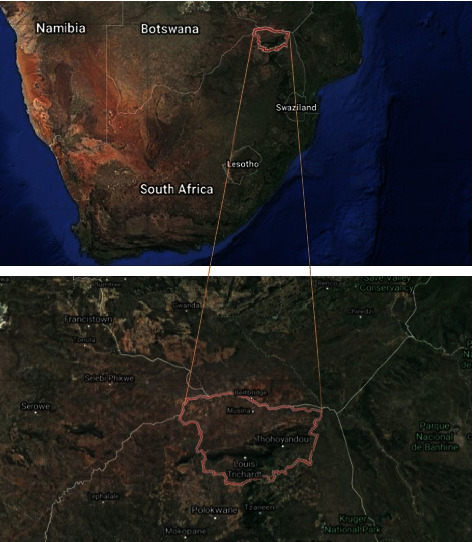
Location of the Vhembe district.

The study area is located in the Vhembe district municipality in the northern part of the Limpopo province. The district lies between latitude 22°, 45′, 17″S and longitude 30°, 12′, 37″E (see Figure 1). The population of the Vhembe district municipality was estimated to be 1,393,949 in 2016 (STATSSA, 2011) within an area of 25,597 km2, which is at a height of 1206 m above sea level. This area receives about 600 mm of rainfall annually. The Vhembe district receives its highest rainfall in November and January. During the winter seasons, this area receives a very low amount of rainfall in July and August. January is observed as the wettest month with an annual rainfall of about 420 mm. July is the driest month with only about 2 mm rainfall. Annually, an average temperature of about 24.6°C is received with a minimum average of 18.9°C in June, and a maximum average of 28.2°C in January. An average annual relative humidity of about 77.4% is recorded in the study area. The area is drained by the famous River Limpopo and its tributaries.

### 2.2. Data sets

Since 1998, the malaria control programme located in Tzaneen, Limpopo, has collected daily malaria case data that are positively confirmed either by microscopy or Rapid Antigen Detection Tests (RDT) at all primary health care facilities and laboratories, which also include private clinics and hospitals. All notified parasitologically confirmed infections are routinely verified, collected, and entered into a computerized malaria information system (MIS) by malaria control programme officers who visit the various facilities twice a week. The data set was aggregated monthly to the district level. For this study, only localized cases of malaria from 1998 to 2017 (57,974 cases and 69% of total malaria cases) were considered.

## 3. Methods

Statistical and spatial analyses were performed on the malaria and spatial data sets using R and ArcGIS 10.4 software. Statistical analysis was performed to determine if there were significant relationships between dependent and independent variables to establish potential malaria case mortality rate determinants. Spatial analysis, which includes buffering and nearness neighbour analysis (NN), was performed on the spatial data such as water bodies, roads, and rivers. Relevant variables were plotted on geographical maps to interpret qualitative information with regard to malaria case mortality rates and potential determinants.

The primary analytical tests and techniques used to analyse the data were normality tests, correlations, and frequency tests. Pallant [14] states that the correlation coefficient from Pearson's product moment correlation is only applicable when applied to data that are normally distributed. Based on the premise that Spearman's rank correlation coefficient analysis procedure does not assume any linearity or normality, Spearman's rank correlation coefficient is applicable for relationship analysis of data that are not normally distributed or linear. In order to ensure consistency across this relationship analysis, the primary correlation coefficient used was Spearman's rank correlation coefficient, which allowed the same correlation analysis to be applied across all variables, whether normally distributed or not.

The Spearman correlation coefficients (*r*) were interpreted according to [15] guidelines that state that *r* = 0.5–1.0 indicates a large relationship, *r* = 0.3–0.49 indicates a medium relationship, and *r* = 0.1–0.29 indicates a small relationship. In addition to the Spearman's correlation coefficient, the findings illustrate Kendall's tau coefficient and Pearson's product moment correlation to ensure that no abnormalities existed within the data. All correlation coefficients were interpreted against a certainty value (*p* value) based on the following principle: A *p* value <0.05 was considered to indicate statistical significance of the respective correlation coefficient, while a *p* value >0.05 rendered the respective correlation coefficient not significant. All survey results were first tested for consistency, normality, and linearity in order to select the relevant approaches for analysis on intervariable correlations [16].

The records included consolidated malaria infection reports from 263 medical facilities servicing residents from 850 source locations (villages and formal neighbourhoods). The total number of records amounted to 57,974, of which 25,943 were female patients 32,020 were male patients, and 11 records did not indicate gender. Of importance to note is that the data set only included malaria cases that were captured by medical facilities and did not include unreported cases in isolated areas.

The entire target population was required to be sampled to obtain as much information from the data as possible. Due to the large data set but the limited variance of the studied malaria mortality rates, a maximum variation sampling technique combined with a stratified sampling approach was used. Maximum variation sampling is a nonprobability, purposive sampling technique, which allows for a comparison to be made of the characteristics of the most differing samples in a data set [9, 15–17].

**Figure 2:**
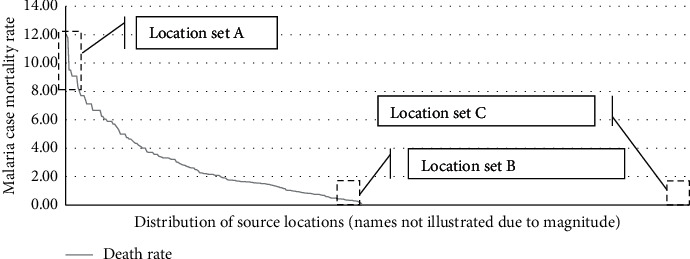
Malaria case mortality rates across the selected source locations.

The target population was divided into three different sections based on malaria case mortality rates, and within these three groups, the top 10 areas were selected for the analysis (which resulted in the selection of 10 source locations from the highest case mortality rate groups, 10 from the middle group, and 10 from the lowest case mortality rate group). This approach ensured that the most significant variance of case mortality rates within the population were compared with variables among the most differing cases [9, 15].  Figure 2 illustrates a graphical illustration of the source locations that were sampled.

The three source locations selected for the analysis (Location set A, B, and C) are described below.

Location set A consists of the 10 locations in the data set with the highest case mortality rates. The case mortality rates ranged between 8% and 12%, and the details are listed in Table 1.

Location set B consists of the 10 locations in the data set with the lowest case mortality rates among all locations that reported fatalities. The case mortality rates in this group ranged between 0.15% and 0.35%, and the details are listed in Table 2.

Location set C consists of the 10 locations in the data set with the highest number of infections and no reported malaria deaths. The case mortality rates in this group are 0% and the number of infections ranged between 171 and 304. The details are illustrated in Table 3.

The dependent variable, as described in Tables 1–3, was malaria case mortality rates, against which all other independent variables were measured to determine potential correlations across various source locations of reported malaria infections within the data set. The malaria case mortality rate was calculated by determining the total number of infections reported in a specific source location (villages, neighbourhoods, etc.) and measuring the total against the number of fatalities from infections in that specific location. In a simple equation,

(1) $$\begin{array}{c} \text{Malaria case mortality rate}=\frac{\text{Number of fatal malaria cases}}{\text{Total malaria cases}}. \end{array}$$

The independent variables that were investigated against the dependent variables were divided into four main socioeconomic categories relating to housing locality and demographics; the number of medical facilities used; the number of infections reported; the distance to medical facilities; and the exposure to significant water bodies.

The malaria data for this study were collated by the malaria control programme at the Limpopo Provincial Department of Health and were obtained from the South African Weather Service through its collaborative research with the University of Pretoria's Institute for Sustainable Malaria Control. No other personal data of patients were collected and no interviews were conducted.

## 4. Results

The data collection and research focused on the 30 locations (illustrated in Tables 1–3) and the correlation between the fixed factors: malaria case mortality rates between 1998 and 2017, and the variable factors.

Table 4 illustrates the outcome of the data collection for the dependent and independent variables in the 30 source locations that made up the study sample.

**Figure 3:**
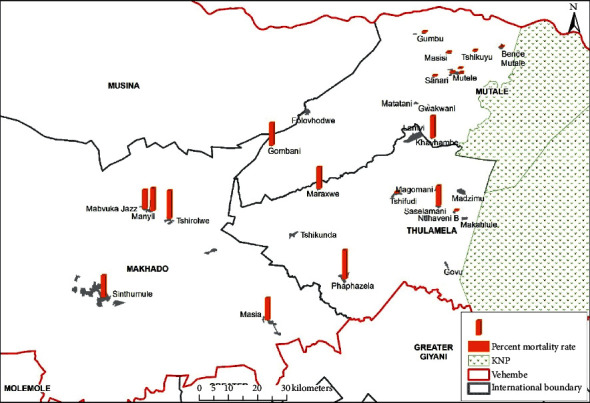
Case mortality rates across the source locations in the study sample.

The findings from the correlation investigation between the results in Table 4 and the malaria case mortality rates are discussed below (see Figure 3 for spatial overview of case mortality rates).

### 4.1. The Correlation between the Number of Medical Facilities Used by the Source Locations and the Malaria Case Mortality Rates

**Figure 4:**
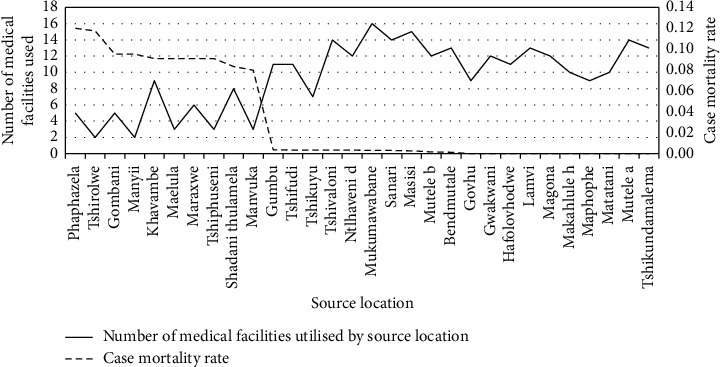
Number of medical facilities used by the source locations and the malaria case mortality rates.

This correlation analysis focused on individual malaria infection source locations and determined the number of medical facilities that individuals from these locations used during the study timeframe. The number of facilities was compared with the malaria case mortality rates to determine the relationship between the two factors (see Figure 4). The correlation analysis illustrated a Spearman's rank correlation coefficient of −0.7952 with a *p* value of 0.0000.

### 4.2. The Correlation between the Maximum Distance from the Medical Facilities Used and the Malaria Case Mortality Rates

**Figure 5:**
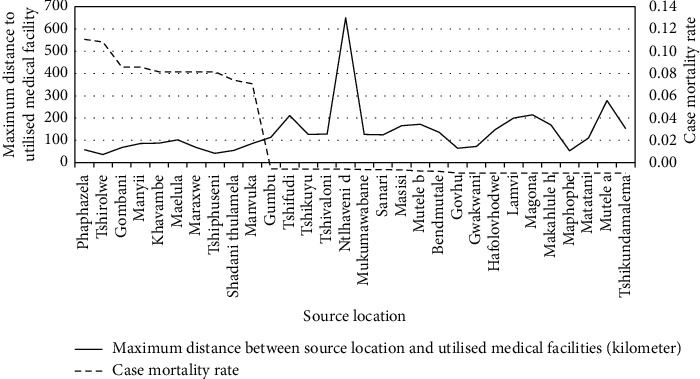
Maximum kilometer travel distance to medical facilities used across the source locations.

The correlation analysis focused on individual malaria infection source locations and determined the maximum distance that individuals in this area travelled to the medical facilities. The maximum distance between source location and the medical facility used was compared with the malaria case mortality rates to determine the relationship between the two factors (see Figure 5). The correlation analysis illustrated a Spearman's rank correlation coefficient of −0.6723 with a *p* value of 0.0000.

**Figure 6:**
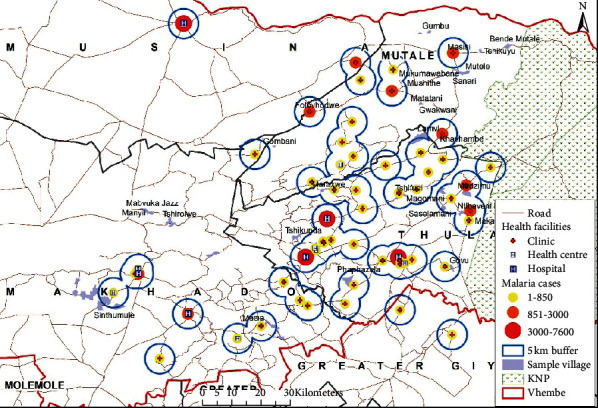
Malaria case source locations within a 5 km buffer of medical facilities.

**Figure 7:**
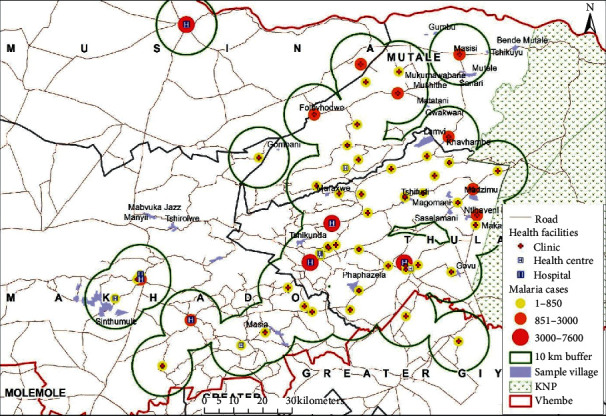
Malaria case source locations within a 10 km buffer of medical facilities.

**Figure 8:**
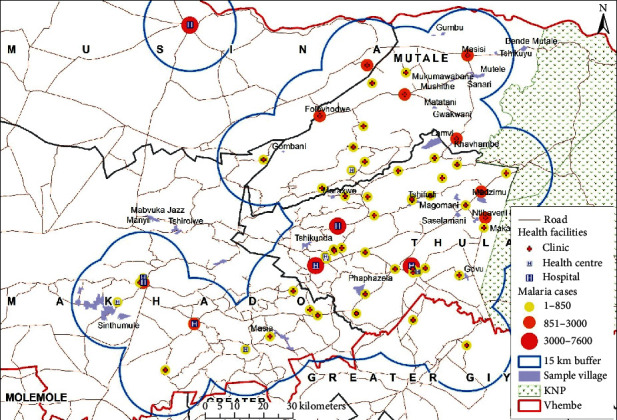
Malaria case source locations within a 15 km buffer of medical facilities.

The distance between medical facilities across the studied source locations have also been plotted on geographical maps (see Figures 6–8) and the spatial analysis supported the statistical findings, with an exception of the following three source locations: Tshirolwe, Manyii, and Manvuka.

### 4.3. The Correlation between the Minimum Distance from the Medical Facilities Used and the Malaria Case Mortality Rates

**Figure 9:**
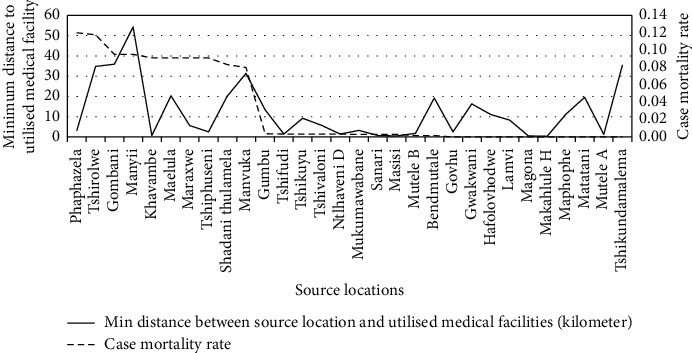
Minimum kilometer travel distance to medical facilities across the source locations.

This correlation analysis focused on individual malaria infection source locations and determined the minimum distance that individuals travelled to the medical facilities used. The minimum distance between the source location and the medical facility was compared with the malaria case mortality rate to determine the relationship between the two factors (see Figure 9). The correlation analysis illustrated a Spearman's rank correlation coefficient of 0.3816 with a *p* value of 0.0375.

Similar to the maximum distance factor, the spatial analysis supported the minimum distance statistical findings, with an exception of the following three source locations: Tshirolwe, Manyii and Manvuka (see Figures 6–8).

### 4.4. The Correlation between the Mean Distance from the Medical Facilities Used and the Malaria Case Mortality Rates

**Figure 10:**
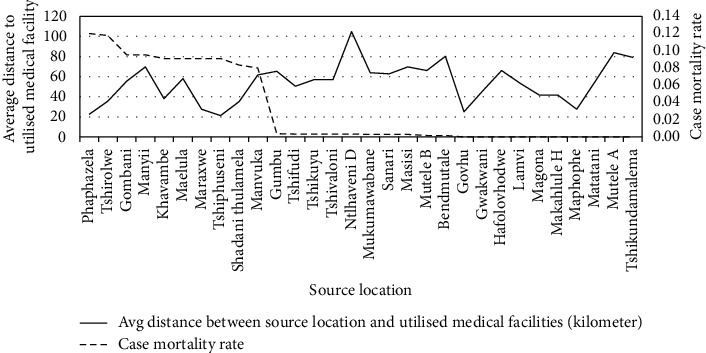
Average kilometer travel distance to the medical facilities across the source locations.

This correlation analysis focused on individual malaria infection source locations and determined the mean distance that individuals travelled to the medical facilities used. The mean distance between the source location and the medical facility was compared with the malaria case mortality rate to determine the relationship between the two factors (see Figure 10). The correlation analysis illustrated a Spearman's rank correlation coefficient of −0.3990 with a *p* value of 0.0289.

Similar to the maximum distance factor, the spatial analysis supported the mean distance statistical findings, with an exception of the following three source locations: Tshirolwe, Manyii, and Manvuka (see Figures 6–8).

### 4.5. The Correlation between the Distance from Significant Water Bodies and the Malaria Case Mortality Rates

**Figure 11:**
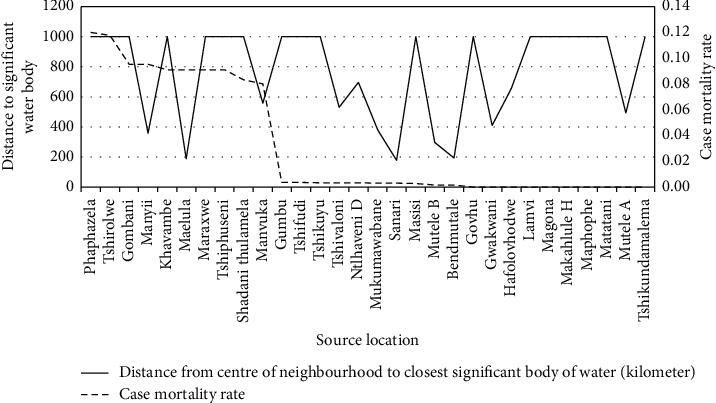
Distance between the source locations and significant bodies of water.

This correlation analysis focused on individual malaria infection source locations and determined the distance between the source location and the nearest significant water body. This distance was compared with the malaria case mortality rate to determine the relationship between the two factors (see Figure 11). The correlation analysis illustrated a Spearman's rank correlation coefficient of 0.1320 with a *p* value of 0.4869.

**Figure 12:**
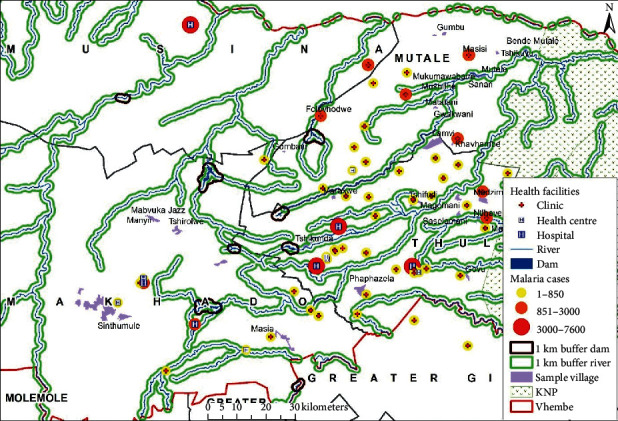
Malaria case source locations within a 1 km buffer of significant water bodies.

The related spatial analysis supported the statistical findings that suggested that proximity to water bodies does not have a significant positive relationship with malaria case fatality rates (see Figure 12).

### 4.6. The Correlation between the Number of Infections Reported by the Source Locations and the Malaria Case Mortality Rates

**Figure 13:**
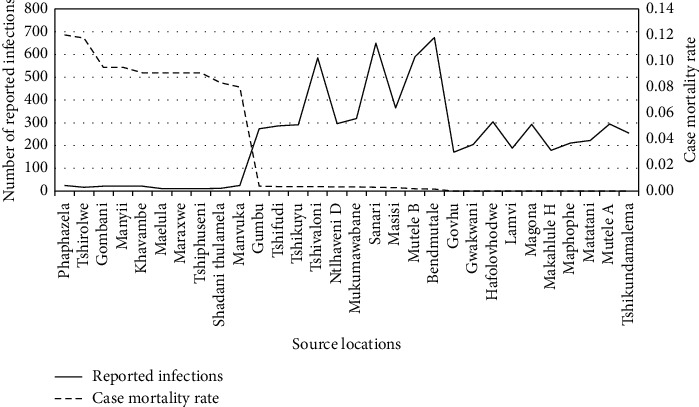
Number of infections reported across the source locations and the malaria case fatality rates.

This correlation analysis focused on individual malaria infection source locations and determined the number of malaria cases reported in the study timeframe. The number of cases reported was compared with the malaria case mortality rates to determine the relationship between the two factors (see Figure 13). The correlation analysis illustrated a Spearman's rank correlation coefficient of −0.7890 with a *p* value of 0.0000.

**Figure 14:**
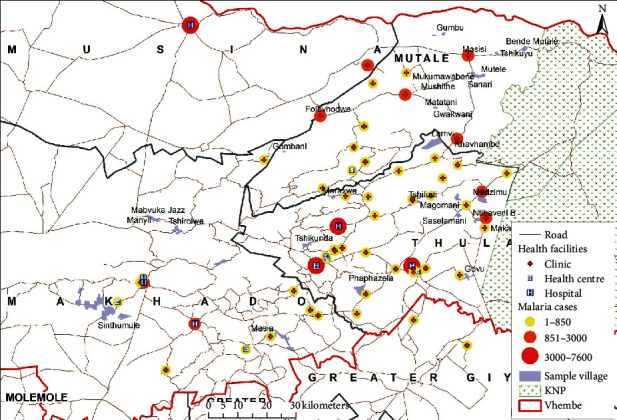
Investigated medical facilities and the total number of reported cases within the study timeframe.

The above mentioned data have also been plotted on a geographical map and the spatial analysis could be considered to support the statistical findings that areas with lower numbers of reported malaria cases are more likely to have high case mortality rates (see Figure 14).

## 5. Discussion

### 5.1. The Correlation between the Number of Medical Facilities Used by the Source Locations and the Malaria Case Mortality Rates

The statistical analysis indicated a strong to a very strong negative relationship between the two variables, with the Spearman's rank correlation coefficient indicating a negative correlation coefficient of −0.7952 with a *p* value of 0.0000. This suggests that if all other variables are considered equal, the number of medical facilities available to a specific source location has a large, negative relationship with the malaria case mortality rates in an area.

The data suggest that there is a very strong likelihood that an increase in the number of medical facilities accessible to a specific source location could lead to a significant decrease in the source location's malaria case mortality rates. It is important to note that these results were calculated only on the number of medical facilities used, but this number could have been influenced by various other factors such as access to transportation, financial means to visit medical facilities, and willingness to seek treatment, for example.

### 5.2. The Correlation between the Maximum Distance from the Medical Facilities Used and the Malaria Case Mortality Rates

The statistical analysis of the results across the three correlation theories indicated a medium to strong negative relationship between the two variables, with the Spearman's rank correlation coefficient indicating a negative correlation coefficient of −0.6723 with a *p* value of 0.0000. This suggests that if all other variables are considered equal, the maximum distance between the medical facilities that the patients used and the source location has a large, negative relationship with the malaria case mortality rates in an area.

The data suggest that there is a likelihood that an increase in the maximum distance between the medical facilities that the patients used and the source location could lead to a significant decrease in the source location's malaria case mortality rates. This result is contrary to the researchers' assumptions that closer access to medical facilities was expected to decrease malaria case mortality rates. The results indicate there could be additional factors that need to be investigated. One possible explanation that should be investigated is that further maximum distances travelled to medical facilities could indicate that patients had financial or other means to access more advanced medical services at larger facilities and, as a result, this translated into smaller case mortality rates.

### 5.3. The Correlation between the Minimum Distance from the Medical Facilities Used and the Malaria Case Mortality Rates

The statistical analysis of the results across the three correlation theories indicated a medium positive relationship between the two variables, with the Spearman's rank correlation coefficient indicating a positive correlation coefficient of 0.3816 with a *p* value of 0.0375. This suggests that if all other variables are considered equal, the minimum distance between the medical facilities used and the source location has a medium, positive relationship with the malaria case mortality rates in an area.

The data suggest that there is a likelihood that a decrease in the minimum distance between the medical facilities used and the source location could lead to a potential decrease in the source location's malaria case mortality rates. This result is in line with the researchers' assumptions that closer access to medical facilities was expected to decrease malaria case mortality rates. The results could indicate that immediate or lower than average access times to medical facilities could decrease malaria case mortality rates.

### 5.4. The Correlation between the Mean Distance from Medical Facilities Used and the Malaria Case Mortality Rates

The statistical analysis of the results across the three correlation theories indicated a medium negative relationship between the two variables, with the Spearman's rank correlation coefficient indicating a negative correlation coefficient of −0.3990 with a *p* value of 0.0289. This suggests that if all other variables are considered equal, the average distance between the medical facilities that patients used and the source location has a medium, negative relationship with the malaria case mortality rates in an area.

The data suggest that there is a likelihood that an increase in the average distance between the medical facilities used and the source location could lead to a significant decrease in the source location's malaria case mortality rate. This result is contrary to the researchers' assumptions that closer access to medical facilities was expected to have a negative impact on malaria case mortality rates. The results indicate that there may be additional factors to be investigated. One possible explanation that should be investigated is that the further average distances travelled to medical facilities could indicate that patients had financial or other means to access more advanced medical services at larger facilities and, as a result, that translated into smaller case mortality rates.

### 5.5. The Correlation between the Distance from Significant Water bodies and the Malaria Case Mortality Rates

The statistical analysis of the results across the three correlation theories indicated a potentially small positive relationship between the two variables, with the Spearman's rank correlation coefficient indicating a positive correlation coefficient of 0.1320 with a *p* value of 0.4869. The potential correlation is however not statistically significant due to the 48.6% probability that the correlation coefficient is coincidental.

The statistical and spatial analyses suggest that there is no significant correlation between the malaria case mortality rate in an area and source location distance to significant water bodies. It is important to note that these results only considered malaria case mortality rates. It should be further investigated if the distance to significant water bodies in Vhembe has an impact on the number of malaria infections.

### 5.6. The Correlation between the Number of Infections Reported by the Source Locations and the Malaria Case Mortality Rates

The statistical analysis of the results across the three correlation theories indicated a strong to a very strong negative relationship between the two variables, with the Spearman's rank correlation coefficient indicating a negative correlation coefficient of −0.7890 with a *p* value of 0.0000. This suggests that if all other variables are considered equal, the number of malaria infections in a specific source location has a large, negative relationship with the malaria case mortality rates in an area.

The data suggest that there is a very strong likelihood that an increase in the number of reported malaria infections in a specific source location could lead to a decrease in the source location's malaria case mortality rate. It is important to note that these results were calculated only on the number of malaria infections reported and were not correlated with population numbers in the various source locations. Investigations should be undertaken on the following potential theories to further explain this finding:

- Lower numbers of infections in source locations could result in populations that are uneducated in malaria treatment, resulting in higher case mortality rates.
- Lower numbers of infections in source locations reflect smaller source location populations, which could result in limited societal support, resulting in higher case mortality rates.

Lower numbers of infections in source locations reflect smaller populations that are socioeconomically segregated from larger, more advanced communities with easier access to medical facilities, which could result in higher case mortality rates.

## 6. Conclusion

The statistical analysis indicated a significant negative correlation between case mortality rates and the number of medical facilities used by the sample source locations, numbers of infections reported, and the maximum and mean distances travelled to the medical facilities used. This suggested that malaria patients from larger communities with financial or other means to access more advanced or a bigger variety of medical facilities had a significantly lower risk of mortality. The negative correlation between average distances travelled to medical facilities and case mortality rates could also suggest that patients who were willing or educationally informed to seek medical assistance during malaria infections, despite long travel distances, had a significantly higher chance of survival.

The two independent variables that indicated the strongest correlations to malaria case fatality rates were the number of medical facilities used by source locations followed by the number of infections reported during the study timeframe. The negative correlation with the number of medical facilities used could suggest that malaria patients from larger communities, those who had access to a larger variety of medical facilities, had a significantly higher survival rate than patients from smaller communities. Although the negative correlation with the number of infections reported supports this statement, it also means that communities with high numbers of infections have improved malaria preparedness in terms of readily available resources to treat malaria infections and prevent related deaths.

The analysis further indicated a positive correlation between the minimum distances travelled to medical facilities and case mortality rates, suggesting that although longer maximum and average travelling distances had a negative correlation, medical facilities situated in the communities could have a positive impact on reducing case mortality rates. This could suggest that communities with socioeconomic means to travel to significantly further medical facilities had lower case mortality rates; communities with medical facilities in significantly close proximity also indicated lower case mortality rates.

Proximity to significant water bodies was not found to have any significant impact on malaria case mortality rates; however, it is suspected that it could have an impact on the number of infections.

The research findings suggest that South African authorities could reduce malaria case mortality rates in the Vhembe district by improving the local communities' access to more advanced health care facilities and a larger variety of health care facilities. This could be accomplished through two primary approaches:

- Physically enabling the communities to access a larger network of health care facilities, either through subsidized or improved public transport systems, increased economic development in the regions, improved road networks, or other measures that could decrease the physical and financial barriers that certain communities experience with regard to accessing health care facilities.
- Socially increasing local communities' willingness to access more advanced health care facilities, which could be achieved through increased educational campaigns and activities to increase community networks and malaria treatment awareness.

In addition, the findings suggest that the distribution of medical treatment facilities in direct proximity of communities could result in decreased malaria case mortality rates.

In terms of future research, the research findings suggest that patients from various source locations in the Vhembe district prefer to use medical facilities that are significantly further away than the medical facilities closest to them, and this decreases the malaria case mortality rates. Further research into understanding the drivers behind the medical facility preferences and medical-seeking behaviour in general in the Vhembe district could enable South African authorities to drive even more effective malaria management principles.

## Figures and Tables

**Table 1:** Location set A.

Source location	No. of malaria cases	No. of malaria deaths	% case mortality rate
Phaphazela	25	3	12.00
Tshirolwe	17	2	11.76
Manyii	21	2	9.52
Gombani	21	2	9.52
Maelula	11	1	9.09
Tshiphuseni	11	1	9.09
Khavambe	22	2	9.09
Maraxwe	11	1	9.09
Shadani	12	1	8.33
Manvuka	25	2	8.00

**Table 2:** Location set B.

Source location	No. of malaria cases	No. of malaria deaths	% case mortality rate
Tshifudi	287	1	0.35
Tshikuyu	292	1	0.34
Tshivaloni	585	2	0.34
Ntlhaveni d	297	1	0.34
Mukumawabane	319	1	0.31
Sanari	649	2	0.31
Masisi	366	1	0.27
Gumbu	273	1	0.37
Mutele b	590	1	0.17
Bendmutale	674	1	0.15

**Table 3:** Location set C.

Source location	No. of malaria cases	No. of malaria deaths	% case mortality rate
Hafolovhodwe	304	0	0.00
Mutele a	295	0	0.00
Magona	293	0	0.00
Tshikundamalema	254	0	0.00
Matatani	222	0	0.00
Maphophe	211	0	0.00
Gwakwani	204	0	0.00
Lamvi	188	0	0.00
Makahlule h	179	0	0.00
Govhu	171	0	0.00

**Table 4:** Overview of socioeconomic factors per source location in the study sample.

Source location	Number of medical facilities used by location	Maximum kilometer distance between location and facilities	Minimum kilometer distance between location and facilities	Average kilometer distance between location and facilities	Distance (kilometer) from the centre of neighbourhood to the closest significant body of water
Phaphazela	5	58.6	3	22.56	>1000
Tshirolwe	2	36.2	34.9	35.55	>1000
Gombani	5	66.9	36	55.36	>1000
Manyii	2	84.9	54.3	69.6	359
Khavambe	9	87.8	0.65	37.91	>1000
Maelula	3	102	20.4	57.9	189
Maraxwe	6	67.3	5.7	27.48	>1000
Tshiphuseni	3	41.6	2.5	21.23	>1000
Shadani thulamela	8	55	20.2	35.13	>1000
Manvuka	3	86.3	31.3	61.7	557
Gumbu	11	114	13.4	65.49	>1000
Tshifudi	11	211	1.5	50.27	>1000
Tshikuyu	7	126	9.2	56.99	>1000
Tshivaloni	14	128	5.8	56.81	531
Ntlhaveni d	12	650	1.5	104.76	695
Mukumawabane	16	127	3.3	63.7	381
Sanari	14	124	0.7	62.82	178
Masisi	15	165	0.55	69.63	>1000
Mutele b	12	172	1.8	66.08	298
Bendmutale	13	136	19.2	80.35	193
Govhu	9	63.9	2.5	24.97	>1000
Gwakwani	12	72.7	16.4	46.06	411
Hafolovhodwe	11	148	11	65.97	656
Lamvi	13	200	8.4	53.19	>1000
Magona	12	214	0.6	41.69	>1000
Makahlule	10	168	0.35	41.61	>1000
Maphophe	9	53.6	11.3	27.47	>1000
Matatani	10	110	19.6	55.34	>1000
Mutele	14	278	1.3	83.76	493
Tshikundamalema	13	153	35.5	79.19	>1000

## Data Availability

The malaria data reported in this manuscript have been sourced from the provincial Integrated Malaria Information System (IMIS) of malaria control programme in the Limpopo Provincial Department of Health and were obtained from the South African Weather Service (SAWS) through its collaborative research with the University of Pretoria Institute for Sustainable Malaria Control (UP ISMC). Data can be accessed with written permission after being requested from the corresponding author.
